# False positive PCR detection of *Tropheryma whipplei *in the saliva of healthy people

**DOI:** 10.1186/1471-2180-7-48

**Published:** 2007-05-29

**Authors:** Jean-Marc Rolain, Florence Fenollar, Didier Raoult

**Affiliations:** 1 Université de la Méditerranée, Unité des Rickettsies, CNRS UMR 6020, IFR 48, Faculté de Médecine, 27 Bd Jean Moulin, 13385 Marseille cedex 05, France

## Abstract

**Background:**

*Tropheryma whipplei*, the agent of Whipple's disease (WD), has been recently isolated and the genomes of two isolates have been fully sequenced. Previous diagnosis tools for the diagnosis of the disease used sequence analysis of the 16S rRNA gene. Using this target gene, the high percentage of detection of the bacterium in saliva of healthy people was in contrast to the negative results obtained with specific target genes. The aim of our study was to compare previously published primers targeting the 16S rRNA gene to real-time PCR with Taqman* probes targeting specific repeat genes only found in the genome of *T. whipplei *in a series of 57 saliva from healthy people.

**Results:**

Although the specific real-time PCR assays with both primers and probes were negative for all the samples, 13 out of 57 samples were positive with different primers previously reported targeting the 16S rRNA gene. Among the positive samples, 8 yielded a 231-bp sequence that was 99.1% identical to that of *Actinomyces odontolyticus*, 2 yielded a 226-bp that was 99.6% identical to that of *A. turicensis*, and 3 yielded a 160-bp sequence that was 98.5% identical to that of *Capnocytophaga gingivalis*. We found that the *C. gingivalis *and *A. odontolyticus *16S rRNA sequences obtained in our study share more than 80% homology with the corresponding 16S rRNA sequences of the *T. whipplei *genomes especially at 5' and 3' end.

**Conclusion:**

Asymptomatic carriers of *T. whipplei *in saliva may exist but their prevalence is much lower than those previously reported. Testing the specificity of designed primers is critical to avoid false positive detection of *T. whipplei*. In atypical case we recommend to test two different specific target genes before concluding.

## Background

*Tropheryma whipplei*, the agent of the Whipple's disease (WD), has been recently isolated and cultured [1] and is classified within the *Actinomycetes *clade [2]. On the basis of sequence analysis of the 16S rRNA gene, several diagnostic PCR assays targeting various parts of this gene have been reported and PCR has become an important diagnostic tool [2]. One of the important limits of PCR targeting the 16S rRNA gene is its specificity. Indeed, positive PCR results have been found in specimens from people without the classic clinical or histological features of WD. In one series, *T. whipplei *DNA was apparently detected in 25 out of 38 waste-water samples obtained from five different sewage treatment plants in Southwest Germany [3], and in 13.3% of either duodenal biopsies specimens or gastric juice specimens from 105 patients with no clinical signs of WD [4]. Similarly, in a random sample of 40 healthy people, Street *et al*. found that 35% had *T. whipplei *DNA detected in their saliva [5]. Dutly *et al*. have also reported the presence of *T. whipplei *DNA in saliva of healthy people [6]. All these data were obtained using primers targeting a part of the 16S rRNA gene. In contrast, using specific primers of the 16S-23S ribosomal DNA intergenic spacer (ITS) [7] and primers targeting a 650-bp fragment of the β-subunit of the RNA polymerase gene (*rpoB*) [8], 100 saliva specimens from people without suspicion of WD failed to yield a significant PCR product signal [9]. Maiwald *et al*. also reported that *T. whipplei *occurs only rarely in intestinal mucosa that lacks histopathologic evidence of WD [10]. These conflicting and confusing data emphasize the need for additional information on the prevalence of *T. whipplei *in healthy people, especially in saliva. For this purpose we have compared in this study the use of specific target genes repeated sequences present in seven copies in the genome of *T. whipplei *[11] in a real-time PCR with Taqman* probes, with the previously published primers targeting the 16S rRNA gene [5,6,12] in saliva from healthy people.

## Results

**Figure 1:**
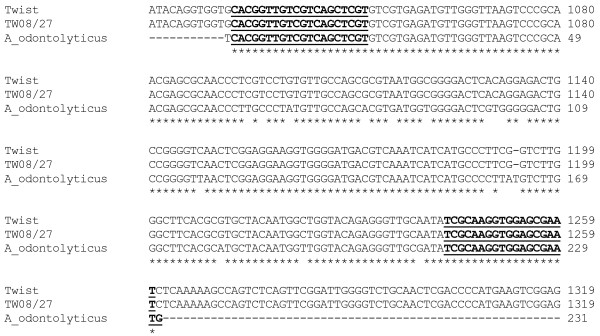
Sequence alignments of the partial sequence of 16S rRNA gene of *T. whipplei *strain Twist, TW08/27 and *A. odontolyticus *amplified from saliva of healthy people in this study using the primers from Dutly *et al*. and Ramzan *et al*. that are in bold and underlined.

**Figure 2:**
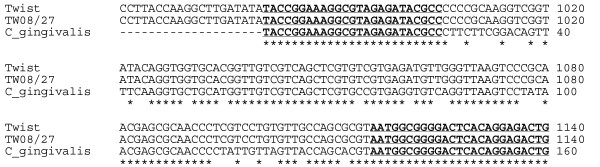
Sequence alignments of the partial sequence of 16S rRNA gene of *T. whipplei *strain Twist, TW08/27 and *C. gingivalis *amplified from saliva of healthy people in this study using the primers from Street *et al*. that are in bold and underlined.

The sensitivity of our real-time PCR assay was of 1 DNA copy of standard control DNA. Specificity was verified using DNA extracted from 40 bacterial strains as previously described [11]; none of the DNA yield a positive signal for the two sets of primers and probes. Although the LightCycler PCR results with both primer pairs and probes were negative for all the saliva, 10 and 3 out of 57 samples were positive with the primers used by Dutly *et al*. and Ramzan *et al*. [6,12] and from Street *et al *[5], respectively. Among the 10 positive samples using the primers of Dutly *et al*., 8 yielded a 231 bp sequence that was 99.1% identical to that of *Actinomyces odontolyticus *(Genbank accession number AJ234047) and 2 yielded a 226 bp that was 99.6% identical to that of *Actinomyces turicensis *(Genbank accession number X78720). The 3 sequences obtained using the primers from Street *et al*. yielded a 160 bp sequence that was 98.5% identical to that of *Capnocytophaga gingivalis *(Genbank accession number X67608). The 16S rRNA sequences of the two available genomes of *T. whipplei *(Strain Twist, Genbank accession number NC 004572 and strain TW08/27, Genbank accession number 00 4551) were retrieved from the KEGG website [13] and were used for sequence alignments using the ClustalW software [14] with our two original sequences of *A. odontolyticus *(Figure 1) and *C. gingivalis *(Figure 2). The *C. gingivalis *16S rRNA sequence shares 80.6% homology (129/160 bp) and the *A. odontolyticus *16S rRNA sequence shares 91.8% homology (212/231 bp) with the corresponding 16S rRNA sequences of the *T. whipplei *genomes (Figures 1 and 2).

## Discussion

In this study, using the same PCR protocols previously used for the detection of *T. whipplei*, the sequences obtained with primers of the 16S rRNA gene were in all cases false positive PCR results with sequences similar to that of bacteria of the oral cavity and not with *T. whipplei*. A better sensitivity and specificity using repeated sequences instead of the 16S rRNA gene has also been reported in other bacteria such as *Coxiella burnetii *[15].

Thus we believe that such high prevalence of *T. whipplei *DNA described in the saliva of healthy people reported using less specific PCR-based methods targeting the 16S rRNA gene are likely due to false positive amplifications of bacteria of the oral cavity. Therefore positive PCR results using the 16S rRNA gene should be interpreted cautiously and sequencing should be the rule. For example, in the study of Street *et al*., only 6 positive PCR products of 14 patients were sequenced [5] yielding a 100% homology with the 16S rRNA sequence of *T. whipplei*. Since the remaining positive samples were not sequenced it is possible that other bacteria found in the oral cavity such as *Actinomyces *spp. or *C. gingivalis *were the cause of the false positive results. This is particularly important when comparing the sequences in our study with the 16S rRNA sequences of *T. whipplei *with a high percentage of homology of the sequences, especially at both the 5' and 3' ends (Figures 1 and 2). Since we have started to perform the diagnosis of WD in our laboratory, *T. whipplei *DNA has been demonstrated using our PCR assays in saliva from 4 out of 620 (0.6%) patients without WD [9,11,16]. Zinkernagel *et al *have demonstrated that the subgingival plaque may serve as a natural habitat of *T. whipplei *[17]. In this study, using a real-time quantitative PCR assay, authors found also that all samples from saliva were negative [17].

## Conclusion

Asymptomatic carriers of *T. whipplei *in saliva may exist but their prevalence is much lower than those previously reported. Testing the specificity of designed primers is critical to avoid false positive detection of *T. whipplei*. In atypical case we recommend to test two different specific target genes before concluding.

## Methods

Approximately 1 ml of saliva from consenting healthy people working in our institution was collected anonymously and aseptically in sterile tubes during our monthly laboratory meeting. These volunteers were from diverse ethnic background, aged from about 20 to 60 years old and their dental status was not known. Experimental research reported in this study has been approved by the ethical committee of IFR48. DNA extraction was performed on each sample as previously described [7,9]. PCR was performed using either primers and protocols described by Street *et al*. [5] and Dutly *et al*. and Ehrbar *et al*. [4,6] or two sets of primers and Taqman* probes targeting specific repeated sequences from the genome of *T. whipplei *[11,18]. Primers TW27F (5'-TGTTTTGTACTGCTTGTAACAGGATCT-3') and TW182R (5'- TCCTGCTCTATCCCTCCTATCATC-3') and probe 27F-182R (6FAM- AGAGATACATTTGTGTTAGTTGTTACA-TAMRA) and primers TW342F (5'- AGATGATGGATCTGCTTTCTTATCTG-3') and TW492R (5'- AACCCTGTCCTGCACCCC-3') and probe 342F-492R (6FAM- TATGTGTGTTGGTTATATATGGG-TAMRA) were used in this study that amplify a 155-bp and a 150-bp fragment of the repeat sequence of *T. whipplei*, respectively. The sensitivity and specificity of our Taqman* PCR assay was determined using tenfold dilutions of a standard suspension of 10^6 ^*T. whipplei *strain Marseille-Twist and DNA extracted from 40 bacterial strains as previously described [11].

## Authors' contributions

JMR carried out the molecular genetic studies, participated in the sequence alignment and drafted the manuscript. FF participated in the sequence alignment and helped to draft the manuscript. DR participated in the design of the study and coordination and helped to draft the manuscript. All authors read and approved the final manuscript.
